# Supplementary material for: Metastasis of Uveal Melanoma with Monosomy-3 Is Associated with a Less Glycogenetic Gene Expression Profile and the Dysregulation of Glycogen Storage
Source: Cancers (Basel). 2020 Jul 29;12(8):2101. doi: 10.3390/cancers12082101 (PMC7463985; doi:10.3390/cancers12082101)
Supplement: Supplementary file 1 [file cancers-12-02101-s001.pdf]

cancers

MDPI

Article

Metastasis of Uveal Melanoma with Monosomy-3 is Associated with a Less Glycogenetic Gene Expression Profile and the Dysregulation of Glycogen Storage

Siranush Vardanyan, Anton Brosig, Hartmut Merz, Mahdy Ranjbar, Vinodh Kakkassery, Salvatore Grisanti, and Aysegül Tura

Supplementary Materials:

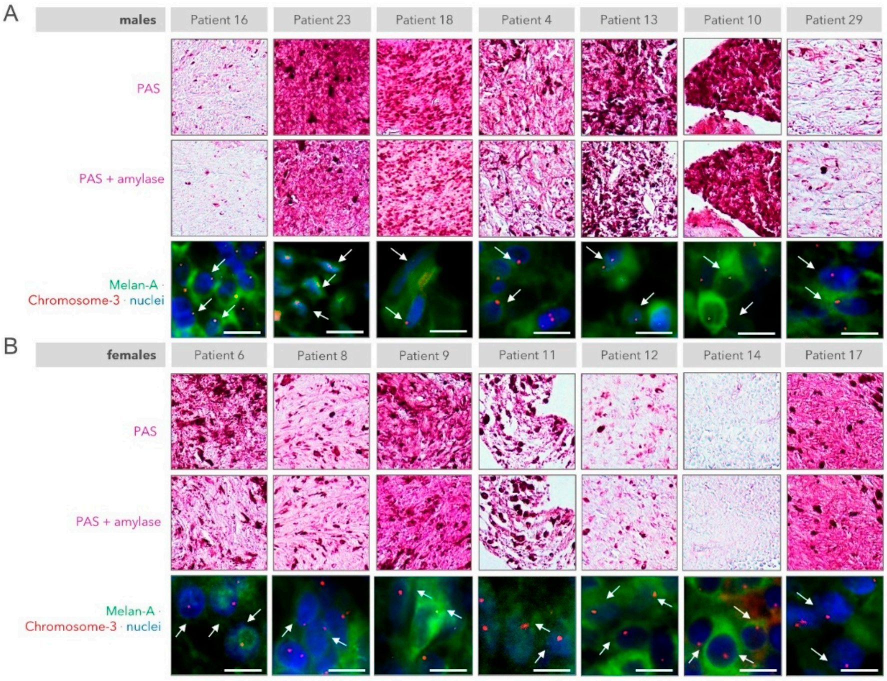

Figure S1. Estimated levels of the amylase-sensitive glycogen in the monosomy-3 tumors of the male versus female patients (n=7 each). **(A)** The amylase pretreatment has led to a slight to moderate weakening in the intensity of the PAS staining in all the male patients except for Patient #29, who did not demonstrate any difference (rightmost panel). **(B)** The tumors of the female patients did not exhibit a noticeable decline in the intensity of the PAS staining except for Patient #6 (leftmost panel), suggesting that the amylase-sensitive glycogen was less abundant in the monosomy-3 tumors of females. All the images of the PAS-stainings were acquired under an original magnification of 100X. The copy number of chromosome-3 (red) together with the melanoma marker protein Melan-A (green) was analyzed by Immuno-FISH. Nuclei were counterstained in blue with DAPI. Arrows indicate several examples of cells with monosomy-3. Scale bars

Table S1. Human genes involved in glycogen synthesis and breakdown (n = 67).

Supplementary Table S1

				not corrected for gene copy number: Tumors with complete information on the copy numbers of chromosome 3p and 3q					corrected for gene copy number: Exclusion of the tumors with aberrations on the other chromosomes that harbor the analyzed genes								
**1. Preparatory steps / Dual regulators of glycogen synthesis* (n=6 genes)**																	
Symbol	NCBI Gene ID	Name	Locus	Expression (not corrected for gene copy number)					Expression (corrected for gene copy number)								References for gene function
				All (n=77)	Disomy-3 (n=38)	Monosomy-3 (n=39)	*p* - value		All		Disomy -3		Monosomy-3		*p* - value		
				Mean ± SD	Median (range)	Median (range)	un-corrected	corrected †	Mean ± SD	n	Median (range)	n	Median (range)	n	un-corrected	corrected †	
GCK	2645	glucokinase	7p13	9.0 ± 3.3	9.0 (0.0-14.7)	9.7 (0.0-14.2)	0.21	1.00	8.9 ± 3.4	71	9.0 (0.0-14.7)	36	9.7 (0.0-14.2)	35	0.19	1.00	[30]
PGM1*	5236	phosphoglucomutase 1	1p31.3	17.0 ± 1.4	15.8 (13.3-18.8)	17.6 (15.9-18.9)	0.00	0.11	17.1 ± 1.4	56	16.9 (13.3-18.8)	32	17.7 (16.8-18.9)	24	0.02	1.00	[16,37,38]
PGM2*	55276	phosphoglucomutase 2	4p14	17.3 ± 0.8	17.0 (15.0-18.3)	17.7 (16.0-19.5)	0.00	0.00	17.2 ± 0.7	68	17.0 (15.0-18.3)	36	17.6 (16.0-18.7)	32	0.00	0.02	[16,38]
PGM3*	5238	phosphoglucomutase 3	6q14.1	15.5 ± 0.8	15.6 (13.6-16.6)	15.6 (13.8-16.8)	0.58	1.00	15.7 ± 0.7	50	15.7 (14.0-16.6)	24	16.0 (14.0-16.8)	26	0.28	1.00	[16,38]
PGM5*	5239	phosphoglucomutase 5	9q21.11	12.4 ±1.2	11.8 (8.3-14.2)	12.8 (10.5-15.5)	0.00	0.02	12.4 ± 1.2	72	11.7 (8.3-14.2)	36	12.8 (10.5-15.5)	36	0.00	0.00	[16,38]
UGP2	7360	UDP-glucose pyrophosphorylase 2	2p15	17.6 ± 0.4	17.6 (16.4-18.7)	17.9 (16.6-18.5)	0.40	1.00	17.6 ± 0.4	67	17.6 (16.4-18.4)	33	17.9 (16.6-18.2)	34	0.30	1.00	[16]
**2. De novo glycogen synthesis (n=5 genes)**																	
Symbol	NCBI Gene ID	Name	Locus	Expression (not corrected for gene copy number)					Expression (corrected for gene copy number)								References for gene function
				All (n=77)	Disomy-3 (n=38)	Monosomy-3 (n=39)	*p* - value		All		Disomy -3		Monosomy-3		*p* - value		
				Mean ± SD	Median (range)	Median (range)	un-corrected	corrected †	Mean ± SD	n	Median (range)	n	Median (range)	n	un-corrected	corrected †	
GBE1	2632	1,4-alpha-glucan branching enzyme 1	3p12.2	16.8 ± 0.7	17.3 (15.6-19.2)	16.5 (15.4-17.1)	0.00	0.00	n.a.	n.a.	n.a.	n.a.	n.a.	n.a.	n.a.	n.a.	[15,16]
GYG1	2992	glycogenin 1	3q24	18.3 ± 0.3	18.4 (17.7-19.2)	18.2 (17.5-18.7)	0.00	0.01	n.a.	n.a.	n.a.	n.a.	n.a.	n.a.	n.a.	n.a.	[15,16]
GYG2	8908	glycogenin 2	Xp22.33	20.8 ± 1.0	21.3 (19.4-23.1)	20.1 (18.9-22.2)	0.00	0.00	20.6 ± 0.9	55	21.2 (19.4-22.7)	29	19.9 (18.9-22.2)	26	0.00	0.00	[15,16]
GYS1	2997	glycogen synthase 1	19q13.33	20.9 ± 0.8	20.7	20.7	0.43	1.00	20.9 ± 0.8	75	20.6	36	20.7	39	0.38	1.00	[15,16,23]

					(18.7-22.7)	(19.7-22.2)					(18.7-22.7)		(19.7-22.2)				
GYS2	2998	glycogen synthase 2	12p12.1	5.5 ± 4.3	0.0 (0.0-9.67)	8.4 (0.0-10.7)	0.00	0.19	5.5 ± 4.3	72	0.0 (0.0-9.7)	37	8.3 (0.0-10.7)	35	0.01	0.38	[15,16,23]

3. Positive regulators of glycogen synthesis (n=19 genes)

Symbol	NCBI Gene ID	Name	Locus	Expression (not corrected for gene copy number)					Expression (corrected for gene copy number)								References for gene function
				All (n=77)	Disomy-3 (n=38)	Monosomy-3 (n=39)	*p* - value		All		Disomy -3		Monosomy-3		*p* - value		
				Mean ± SD	Median (range)	Median (range)	un-corrected	corrected †	Mean ± SD	n	Median (range)	n	Median (range)	n	un-corrected	corrected †	
ATG7	10533	autophagy related 7	3p25.3	16.2 ± 0.4	16.4 (15.8-17.2)	16.0 (14.9-17.0)	0.00	0.00	n.a.	n.a.	n.a.	n.a.	n.a.	n.a.	n.a.	n.a.	[15]
BAP1	8314	BRCA1 associated protein 1	3p21.1	19.0 ± 1.5	20.2 (19.0-21.7)	17.7 (15.9-19.8)	0.00	0.00	n.a.	n.a.	n.a.	n.a.	n.a.	n.a.	n.a.	n.a.	[22]
EPM2A	7957	EPM2A glucan phosphatase, laforin	6q24.3	16.2 ± 0.8	16.7 (15.3-17.7)	16.0 (14.5-17.2)	0.00	0.00	16.5 ± 0.6	50	16.9 (15.6-17.6)	24	16.1 (15.5-16.9)	26	0.00	0.00	[16]
INS	3630	insulin	11p15.5	1.6 ± 4.1	0.0 (0.0-18.4)	0.0 (0.0-12.7)	0.89	1.00	1.7 ± 4.2	68	0.0 (0.0-18.4)	34	0.0 (0.0-12.7)	34	0.90	1.00	[23]
INSR	3643	insulin receptor	19p13.2	16.9 ± 0.4	17.1 (16.3-17.9)	16.7 (16.2-17.7)	0.00	0.00	17.0 ± 0.4	73	17.1 (16.5-17.9)	35	16.7 (16.2-17.7)	38	0.00	0.00	[31]
IRS1	3667	insulin receptor substrate 1	2q36.3	17.0 ± 0.9	17.3 (15.2-18.9)	16.9 (14.0-19.1)	0.01	0.52	17.0 ± 0.8	69	17.3 (15.4-18.9)	32	16.9 (14.0-19.1)	37	0.01	0.43	[39]
IRS2	8660	insulin receptor substrate 2	13q34	19.0 ± 0.6	19.2 (18.5-20.2)	18.7 (17.3-20.2)	0.00	0.00	19.0 ± 0.5	68	19.2 (18.5-20.1)	34	18.8 (17.6-19.7)	34	0.00	0.00	[32]
IRS4	8471	insulin receptor substrate 4	Xq22.3	10.7 ± 3.8	13.6 (0.0-15.7)	10.3 (0.0-13.1)	0.00	0.00	10.7 ± 4.0	55	13.6 (0.0-15.7)	29	10.5 (0.0-13.1)	26	0.00	0.02	[33]
NNAT	4826	neuronatin	20q11.23	12.7 ± 2.5	12.6 (0.0-19.5)	12.7 (9.0-18.6)	0.39	1.00	12.6 ± 2.5	68	12.6 (0.0-19.5)	34	12.7 (9.0-18.6)	34	0.31	1.00	[40]
PPARGC1A	10891	PPARG coactivator 1 alpha	4p15.2	10.1 ± 3.0	11.2 (0.0-15.7)	9.4 (0.0-14.1)	0.01	0.46	10.3 ± 3.1	68	11.2 (0.0-15.7)	36	9.6 (0.0-14.1)	32	0.03	1.00	[41]
PPP1CA	5499	protein phosphatase 1 catalytic subunit alpha	11q13.2	21.0 ± 0.6	21.0 (19.8-22.4)	21.0 (20.2-22.3)	0.32	1.00	20.9 ± 0.6	66	20.9 (19.8-22.1)	31	21.0 (20.2-22.0)	35	0.22	1.00	[15]
PPP1CB	5500	protein phosphatase 1 catalytic subunit beta	2p23.2	19.2 ± 0.6	19.1 (18.1-20.1)	19.4 (17.2-20.5)	0.81	1.00	19.2 ± 0.6	67	19.1 (18.1-20.1)	33	19.3 (17.2-20.0)	34	0.63	1.00	[15]
PPP1CC	5501	protein phosphatase 1 catalytic subunit gamma	12q24.11	18.9 ± 0.4	19.1 (18.1-20.1)	18.7 (18.0-19.7)	0.01	0.46	18.9 ± 0.4	72	19.1 (18.1-20.1)	37	18.7 (18.0-19.6)	35	0.00	0.10	[15,16]
PPP1R3A	5506	protein phosphatase 1 regulatory subunit 3A	7q31.1	0.5 ± 1.8	0.0 (0.0-0.0)	0.0 (0.0-8.61)	0.02	1.00	0.5 ± 1.9	72	0.0 (0.0-0.0)	37	0.0 (0.0-8.6)	35	0.02	1.00	[15,16]

PPP1R3B	79660	protein phosphatase 1 regulatory subunit 3B	8p23.1	17.2 ± 1.1	18.0 (16.1-20.4)	16.5 (15.1-17.7)	**0.00**	**0.00**	17.4 ± 0.8	44	17.9 (16.1-18.7)	29	16.7 (15.8-17.7)	15	**0.00**	**0.00**	[15,16]
PPP1R3C	5507	protein phosphatase 1 regulatory subunit 3C	10q23.32	18.6 ± 1.5	19.6 (15.9-21.4)	17.7 (15.0-19.5)	**0.00**	**0.00**	18.6 ± 1.5	76	19.6 (15.9-21.4)	38	17.7 (15.0-19.5)	38	**0.00**	**0.00**	[15,16]
PPP1R3D	5509	protein phosphatase 1 regulatory subunit 3D	20q13.33	16.6 ± 0.3	16.6 (16.0-18.2)	16.6 (16.1-17.4)	0.92	1.00	16.6 ± 0.3	68	16.6 (16.0-17.1)	34	16.6 (16.1-17.1)	34	0.79	1.00	[15,16]
PRKAG2	51422	protein kinase AMP-activated non-catalytic subunit gamma 2	7q36.1	16.1 ± 0.5	16.2 (14.9-17.0)	16.0 (15.1-17.2)	**<0.05**	1.00	16.1 ± 0.4	71	16.2 (14.9-17.0)	36	16.0 (15.1-16.6)	35	**0.02**	1.00	[15]
TRIM7	81786	tripartite motif containing 7	5q35.3	11.6 ± 1.2	11.8 (9.4-15.6)	11.3 (9.2-13.1)	**0.02**	1.00	11.6 ± 1.1	70	11.8 (9.4-15.6)	34	11.3 (9.2-13.1)	36	0.10	1.00	[16]
4. Negative regulators of glycogen synthesis (n=37 genes)																	
Symbol	NCBI Gene ID	Name	Locus	Expression (not corrected for gene copy number)					Expression (corrected for gene copy number)								References for gene function
				All (n=77)	Disomy-3 (n=38)	Monosomy-3 (n=39)	*p* - value		All		Disomy -3		Monosomy-3		*p* - value		
				Mean ± SD	Median (range)	Median (range)	un-corrected	corrected †	Mean ± SD	n	Median (range)	n	Median (range)	n	un-corrected	corrected †	
AGL	178	amylo-alpha-1, 6-glucosidase, 4-alpha-glucanotransferase	1p21.2	15.0 ± 0.9	14.9 (12.9-16.5)	14.9 (12.9-16.6)	0.97	1.00	15.2 ± 0.8	56	15.0 (13.7-16.5)	32	15.5 (13.8-16.6)	24	0.23	1.00	[15]
AMY1A	276	amylase alpha 1A	1p21.1	1.3 ± 3.0	0.0 (0.0-8.8)	0.0 (0.0-9.4)	0.28	1.00	1.2 ± 2.9	56	0.0 (0.0-8.8)	32	0.0 (0.0-8.3)	24	0.75	1.00	[15,44]
AMY1B	277	amylase alpha 1B	1p21.1	1.0 ± 2.7	0.0 (0.0-9.0)	0.0 (0.0-10.2)	0.67	1.00	1.0 ± 2.8	56	0.0 (0.0-8.6)	32	0.0 (0.0-10.2)	24	0.44	1.00	[15,44]
AMY2A	279	amylase alpha 2A	1p21.1	2.6 ± 4.1	0.0 (0.0-10.2)	0.0 (0.0-11.8)	0.41	1.00	2.4 ± 4.1	56	0.0 (0.0-10.2)	32	0.0 (0.0-11.8)	24	0.17	1.00	[15,44]
AMY2B	280	amylase alpha 2B	1p21.1	12.6 ± 1.6	12.4 (10.1-16.2)	13.0 (6.9-15.5)	0.23	1.00	12.7 ± 1.4	56	12.4 (10.1-16.2)	32	13.2 (10.7-14.8)	24	**0.05**	1.00	[15,44]
CALM1	801	calmodulin 1	14q32.11	19.9 ± 0.4	19.7 (18.9-20.6)	20.2 (19.7-20.9)	**0.00**	**0.00**	19.9 ± 0.4	72	19.7 (18.9-20.6)	36	20.2 (19.7-20.9)	36	**0.00**	**0.00**	[16]
CALM2	805	calmodulin 2	2p21	19.8 ± 0.5	19.5 (18.2-20.1)	20.1 (18.5-21.1)	**0.00**	**0.00**	19.8 ± 0.5	67	19.5 (18.2-20.1)	33	20.1 (18.5-20.6)	34	**0.00**	**0.00**	[16]
CALM3	808	calmodulin 3	19q13.32	21.7 ± 0.6	21.8 (20.8-22.9)	21.6 (20.5-22.7)	0.12	1.00	21.7 ± 0.5	75	21.8 (20.8-22.9)	36	21.6 (20.5-22.7)	39	0.20	1.00	[16]
G6PC	2538	glucose-6-phosphatase catalytic subunit	17q21.31	0.9 ± 2.5	0.0 (0.0-7.3)	0.0 (0.0-9.2)	**0.02**	1.00	1.0 ± 2.5	62	0.0 (0.0-7.3)	28	0.0 (0.0-9.2)	34	**0.05**	1.00	[16]

G6PC2	57818	glucose-6-phosphatase catalytic subunit 2	2q31.1	6.2 ± 4.9	7.3 (0.0-12.3)	9.2 (0.0-15.3)	0.02	1.00	6.3 ± 4.9	70	7.2 (0.0-12.3)	33	9.3 (0.0-15.3)	37	0.03	1.00	[16]
G6PC3	92579	glucose-6-phosphatase catalytic subunit 3	17q21.31	20.3 ± 0.9	20.0 (18.5-21.7)	20.7 (19.2-22.3)	0.00	0.00	20.3 ± 0.9	62	19.8 (18.5-21.7)	28	20.6 (19.2-22.0)	34	0.00	0.02	[16]
GAA	2548	glucosidase alpha, acid	17q25.3	20.3 ± 0.7	20.1 (18.7-21.7)	20.3 (19.4-22.0)	0.02	1.00	20.1 ± 0.7	62	19.7 (18.7-21.7)	28	20.3 (19.4-21.3)	34	0.00	1.00	[15,16]
GANC	2595	glucosidase alpha, neutral C	15q15.1	16.7 ± 0.5	16.6 (14.9-17.3)	16.8 (15.3-17.8)	0.16	1.00	16.7 ± 0.5	72	16.6 (15.4-17.3)	37	16.8 (15.9-17.8)	35	0.20	1.00	[45]
GCG	2641	glucagon	2q24.2	0.4 ± 2.2	0.0 (0.0-16.3)	0.0 (0.0-7.3)	0.52	1.00	0.5 ± 2.3	70	0.0 (0.0-16.3)	33	0.0 (0.0-7.3)	37	0.47	1.00	[46]
GCGR	2642	glucagon receptor	17q25.3	6.6 ± 4.9	7.3 (0.0-14.5)	9.5 (0.0-14.9)	0.02	1.00	6.3 ± 5.0	62	7.2 (0.0-12.8)	28	9.1 (0.0-14.9)	34	0.01	0.99	[46]
GCKR	2646	glucokinase regulator	2p23.3	1.3 ± 3.0	0.0 (0.0-10.1)	0.0 (0.0-9.7)	0.29	1.00	1.1 ± 2.8	67	0.0 (0.0-8.8)	33	0.0 (0.0-9.7)	34	0.02	1.00	[16]
GFPT1	2673	glutamine--fructose-6-phosphate transaminase 1	2p13.3	17.0 ± 0.7	16.6 (15.5-17.4)	17.4 (15.8-18.4)	0.00	0.00	17.0 ± 0.7	67	16.6 (15.5-17.4)	33	17.4 (15.8-18.4)	34	0.00	0.00	[34]
GSK3A	2931	glycogen synthase kinase 3 alpha	19q13.2	19.3 ± 0.7	19.2 (18.2-21.3)	19.2 (18.4-20.5)	0.89	1.00	19.3 ± 0.7	75	19.2 (18.2-21.3)	36	19.2 (18.4-20.5)	39	0.77	1.00	[23]
GSK3B	2932	glycogen synthase kinase 3 beta	3q13.33	17.4 ± 0.3	17.6 (16.8-18.1)	17.3 (16.2-17.8)	0.00	0.00	n.a.	n.a.	n.a.	n.a.	n.a.	n.a.	n.a.	n.a.	[15.23]
INPP5K	51763	inositol polyphosphate-5-phosphatase K	17p13.3	19.2 ± 0.7	19.2 (18.0-21.0)	19.1 (17.5-20.5)	0.82	1.00	19.1 ± 0.7	68	19.2 (18.0-21.0)	35	19.0 (18.0-20.3)	33	0.62	1.00	[47]
NHLRC1	378884	NHL repeat containing E3 ubiquitin protein ligase 1	6p22.3	17.1 ± 1.0	17.1 (10.0-19.0)	17.4 (16.6-18.2)	0.01	0.97	17.2 ± 0.5	36	16.7 (15.7-17.3)	6	17.4 (16.6-17.8)	30	0.01	0.82	[40]
NUDT14	256281	nudix hydrolase 14	14q32.33	19.8 ± 1.0	19.4 (17.8-21.4)	19.8 (18.8-21.9)	0.01	0.94	19.8 ± 0.9	71	19.3 (17.9-21.4)	35	19.8 (18.8-21.7)	36	0.02	1.00	[48]
PCK1	5105	phosphoenolpyruvate carboxykinase 1	20q13.31	7.0 ± 3.8	6.9 (0.0-9.8)	9.4 (6.1-12.8)	0.00	0.00	6.8 ± 3.9	68	6.5 (0.0-9.8)	34	9.3 (6.1-12.8)	34	0.00	0.00	[35]
PFKM	5213	phosphofructokinase, muscle	12q13.11	19.4 ± 0.5	19.4 (17.9-20.2)	19.5 (18.5-20.7)	0.36	1.00	19.4 ± 0.4	72	19.4 (18.1-20.2)	37	19.5 (18.5-20.2)	35	0.57	1.00	[49]
PHKA1	5255	phosphorylase kinase regulatory subunit alpha 1	Xq13.1	14.9 ± 1.9	15.5 (10.9-17.8)	14.6 (9.7-17.4)	0.05	1.00	14.9 ± 1.8	56	15.5 (10.9-17.8)	29	14.6 (10.0-17.0)	27	0.11	1.00	[16]

PHKA2	5256	phosphorylase kinase regulatory subunit alpha 2	Xp22.13	16.8 ± 0.6	17.1 (15.8-18.3)	16.5 (15.3-17.9)	0.00	0.00	16.8 ± 0.6	56	17.1 (15.8-18.3)	29	16.5 (15.3-17.9)	27	0.00	0.03	[16]
PHKB	5257	phosphorylase kinase regulatory subunit beta	16q12.1	16.3 ± 0.8	16.0 (14.4-17.4)	16.7 (15.3-18.1)	0.00	0.03	16.4 ± 0.7	63	16.1 (14.7-17.4)	33	16.9 (15.3-17.7)	30	0.00	0.06	[16]
PHKG1	5260	phosphorylase kinase catalytic subunit gamma 1	7p11.2	13.9 ± 0.8	13.8 (11.9-16.3)	14.1 (12.8-15.5)	0.06	1.00	13.9 ± 0.8	71	13.7 (11.9-16.3)	36	14.1 (12.8-15.5)	35	0.02	1.00	[16]
PHKG2	5261	phosphorylase kinase catalytic subunit gamma 2	16p11.2	17.0 ± 0.6	16.9 (15.8-18.2)	16.9 (16.2-17.9)	0.84	1.00	17.0 ± 0.5	74	16.9 (15.8-18.2)	37	16.9 (16.2-17.9)	37	0.96	1.00	[16]
PPP1R14C	81706	protein phosphatase 1 regulatory inhibitor subunit 14C	6q25.1	8.9 ± 5.2	7.9 (0.0-12.2)	12.6 (9.7-16.4)	0.00	0.00	9.1 ± 5.2	50	7.9 (0.0-12.2)	24	12.7 (9.7-16.4)	26	0.00	0.00	[36]
PRDM8	56978	PR/SET domain 8	4q21.21	9.7 ± 3.4	9.1 (0.0-14.0)	10.9 (0.0-17.9)	0.00	0.06	9.8 ± 3.4	70	9.1 (0.0-14.0)	35	11.1 (0.0-17.9)	35	0.00	0.16	[16,50]
PYGB	5834	glycogen phosphorylase B	20p11.21	21.7 ± 1.0	22.1 (20.1-24.6)	21.3 (19.7-23.2)	0.00	0.29	21.7 ± 1.0	67	22.1 (20.1-24.6)	33	21.3 (20.2-23.0)	34	0.00	0.19	[15,16]
PYGL	5836	glycogen phosphorylase L	14q22.1	21.2 ± 1.3	21.6 (18.8-22.9)	21.3 (16.8-23.7)	0.48	1.00	21.2 ± 1.3	72	21.7 (18.8-22.9)	36	21.4 (16.8-23.5)	36	0.36	1.00	[15,16]
PYGM	5837	glycogen phosphorylase, muscle associated	11q13.1	13.2 ± 1.4	12.3 (10.2-15.1)	13.9 (11.5-16.2)	0.00	0.00	13.2 ± 1.3	66	12.2 (10.6-14.3)	31	13.9 (11.5-16.0)	35	0.00	0.00	[15,16]
RBCK1	10616	RANBP2-type and C3HC4-type zinc finger containing 1	20p13	20.0 ± 0.7	19.9 (18.9-21.8)	19.7 (18.9-21.5)	0.24	1.00	19.8 ± 0.6	67	19.8 (18.9-21.7)	33	19.7 (18.9-20.7)	34	0.29	1.00	[51]
TFEB	7942	transcription factor EB	6p21.1	15.3 ± 0.9	15.5 (13.2-17.3)	15.2 (13.2-17.2)	0.11	1.00	15.2 ± 0.7	36	15.7 (15.0-16.3)	6	15.1 (13.9-17.2)	30	0.04	1.00	[52]
UGDH	7358	UDP-glucose 6-dehydrogenase	4p14	16.8 ± 0.7	16.5 (15.1-18.2)	16.8 (16.0-18.5)	0.03	1.00	16.7 ± 0.6	68	16.5 (15.1-18.2)	36	16.7 (16.0-17.9)	32	0.21	1.00	[53]

n= number; n.a.: not analyzed; NCBI: National Center for Biotechnology Information; TCGA: The Cancer Genome Atlas; SD: standard deviation; UM: Uveal melanoma. *Genes that are involved in both the synthesis and degradation of glycogen.

P - values were determined by the Mann-Whitney U test. † Bonferroni correction.

Genes with a differential expression in monosomy-3-tumors that remained significant after Bonferroni correction were highlighted in colored background. References for gene function are presented in the main text.

Table S2. Genes with a lower expression level in the monosomy-3 tumors regardless of the gene copy number (n = 13),

Supplementary Table S2

Genes with a lower expression level in the monosomy-3 tumors regardless of the gene copy number (n=13) · Median expression (range) in log_2(FPKM-uq) units

Function*	#1-11. De novo glycogen synthesis & positive regulators of glycogen synthesis											#12,13. Negative regulators of glycogen synthesis	
Gene symbol	ATG7	BAP1	EPM2A	GBE1	GYG1	GYG2	INSR	IRS2	IRS4	PPP1R3B	PPP1R3C	GSK3B	PHKA2
Locus	3p25.3	3p21.1	6q24.3	3p12.2	3q24	Xp22.33	19p13.2	13q34	Xq22.3	8p23.1	10q23.32	3q13.33	Xp22.13
Expression (n=80)	16.2	19.5	16.2	16.7	18.2	20.8	17.0	19.0	10.9	17.3	18.5	17.4	16.7
	(14.9-17.2)	(15.9-21.7)	(14.5-17.7)	(15.4-19.2)	(17.5-19.2)	(18.9-23.1)	(16.2-17.9)	(17.3-20.2)	(0.0-15.7)	(15.1-20.4)	(15.0-21.4)	(16.2-18.1)	(15.3-18.3)
Age (n=80)	ATG7	BAP1	EPM2A	GBE1	GYG1	GYG2	INSR	IRS2	IRS4	PPP1R3B	PPP1R3C	GSK3B	PHKA2
≤Median (n=40)	16.2	19.6	16.2	16.8	18.2	20.8	17.0	18.9	11.3	17.3	18.8	17.4	16.9
	(15.3-17.2)	(16.1-21.0)	(14.5-17.5)	(15.4-19.2)	(17.7-19.1)	(19.0-23.1)	(16.2-17.9)	(17.3-20.1)	(0.0-15.7)	(15.1-20.4)	(15.6-21.4)	(16.2-18.1)	(15.5-18.3)
>Median (n=40)	16.2	19.4	16.1	16.6	18.2	20.9	17.0	19.0	10.3	17.2	18.2	17.4	16.6
	(14.9-17.1)	(15.9-21.7)	(14.6-17.7)	(15.6-17.9)	(17.5-19.2)	(18.9-22.9)	(16.2-17.7)	(17.6-20.2)	(0.0-15.3)	(15.2-18.5)	(15.0-21.3)	(16.7-18.1)	(15.3-17.9)
p - value†	0.46	0.89	0.80	0.19	0.72	0.91	0.66	0.79	**0.03**	0.23	0.19	0.49	0.42
Gender (n=80)	ATG7	BAP1	EPM2A	GBE1	GYG1	GYG2	INSR	IRS2	IRS4	PPP1R3B	PPP1R3C	GSK3B	PHKA2
Female (n=35)	16.1	19.6	16.2	16.7	18.2	21.3	17.0	19.1	10.7	17.5	18.6	17.4	16.7
	(14.9-17.1)	(16.1-21.3)	(15.0-17.7)	(15.4-17.7)	(17.5-19.2)	(19.4-23.1)	(16.2-17.9)	(17.3-20.2)	(0.0-15.0)	(15.4-19.1)	(15.6-21.4)	(16.2-18.1)	(16.1-18.0)
Male (n=45)	16.3	19.3	16.1	16.8	18.3	20.6	16.9	18.9	10.9	17.1	18.4	17.4	16.7
	(15.5-17.2)	(15.9-21.7)	(14.5-17.6)	(15.6-19.2)	(17.6-19.1)	(18.9-22.0)	(16.2-17.7)	(17.6-20.2)	(0.0-15.7)	(15.1-20.4)	(15.0-21.3)	(16.7-18.0)	(15.3-18.3)
p - value†	0.18	0.83	0.31	0.87	0.26	**0.00**	0.92	0.39	0.77	0.21	0.40	0.87	0.66
Eye color (n=30)	ATG7	BAP1	EPM2A	GBE1	GYG1	GYG2	INSR	IRS2	IRS4	PPP1R3B	PPP1R3C	GSK3B	PHKA2
Brown (n=15)	16.3	19.8	16.3	16.7	18.2	21.0	17.0	19.1	11.3	17.2	18.5	17.3	17.0
	(15.3-17.2)	(16.6-21.7)	(14.5-17.0)	(15.7-17.6)	(17.7-19.1)	(19.4-23.1)	(16.2-17.9)	(17.3-20.2)	(8.2-14.3)	(15.4-19.1)	(15.6-20.8)	(16.8-18.1)	(16.2-18.0)
Green (n=6)	16.2	19.1	15.7	16.6	18.3	20.6	16.9	19.2	9.9	16.3	17.7	17.5	16.9
	(15.6-16.5)	(16.4-20.0)	(14.9-17.2)	(15.9-17.7)	(17.9-18.9)	(19.0-21.3)	(16.3-17.2)	(18.0-19.6)	(0.0-14.6)	(15.2-20.0)	(16.9-19.7)	(17.0-18.0)	(15.8-17.5)
Blue (n=9)	16.3	19.8	15.9	16.9	18.2	21.3	17.0	19.0	10.9	17.5	18.2	17.3	16.9
	(15.6-17.1)	(16.2-21.3)	(15.0-17.4)	(16.1-17.7)	(18.0-18.5)	(19.5-22.9)	(16.4-17.4)	(18.4-20.0)	(9.2-14.9)	(15.4-18.8)	(17.5-20.9)	(16.9-17.9)	(16.1-17.6)
p - value††	0.54	0.58	0.57	0.90	0.92	0.21	0.85	0.94	0.39	0.68	0.48	0.82	0.76
Largest Basal Diameter (n=74)	ATG7	BAP1	EPM2A	GBE1	GYG1	GYG2	INSR	IRS2	IRS4	PPP1R3B	PPP1R3C	GSK3B	PHKA2
≤13 mm (n=12)	16.3	20.0	16.9	16.9	18.5	21.7	17.1	19.1	11.5	17.5	19.5	17.5	17.1
	(15.6-17.2)	(19.0-21.3)	(14.9-17.7)	(15.4-17.6)	(17.9-19.0)	(19.0-22.7)	(16.3-17.5)	(18.0-19.6)	(8.2-15.0)	(15.7-18.7)	(16.9-20.9)	(16.2-17.8)	(15.8-17.5)
>13 mm (n=62)	16.2	19.0	16.1	16.7	18.2	20.7	17.0	18.9	10.7	17.1	18.3	17.3	16.6
	(14.9-17.1)	(15.9-21.7)	(14-5-17.6)	(15.6-19.2)	(17.5-19.2)	(18.9-23.1)	(16.2-17.9)	(17.3-20.2)	(0.0-15.7)	(15.2-20.0)	(15.0-21.4)	(16.7-18.1)	(15.3-18.3)
p - value†	0.17	**0.00**	**0.03**	0.26	**<0.05**	**0.00**	0.73	0.58	0.34	0.31	**0.02**	0.84	0.25
Tumor thickness (n=76)	ATG7	BAP1	EPM2A	GBE1	GYG1	GYG2	INSR	IRS2	IRS4	PPP1R3B	PPP1R3C	GSK3B	PHKA2
< Median (n=37)	16.1	19.6	16.3	16.8	18.3	21.0	17.1	19.1	10.7	17.3	18.7	17.3	16.7
	(14.9-17.1)	(16.3-21.3)	(14.5-17.7)	(15.4-17.9)	(17.5-19.2)	(19.0-22.7)	(16.2-17.7)	(17.3-20.1)	(0.0-15.3)	(15.3-18.8)	(15.0-21.3)	(16.2-18.1)	(15.3-17.8)
≥ Median (n=39)	16.3	19.3	15.9	16.7	18.2	20.7	17.0	19.0	10.9	17.2	18.2	17.4	16.6
	(15.3-17.2)	(15.9-21.7)	(14.6-17.5)	(15.6-19.2)	(17.6-19.1)	(18.9-23.1)	(16.3-17.9)	(17.9-20.2)	(0.0-15.7)	(15.2-20.0)	(15.6-21.4)	(16.7-18.1)	(15.8-18.3)
p - value†	0.83	0.98	**0.03**	0.76	0.21	0.16	0.48	0.82	0.51	0.92	**0.06**	0.65	0.34

AJCC Stage, clinical (n=80)	ATG7	BAP1	EPM2A	GBE1	GYG1	GYG2	INSR	IRS2	IRS4	PPP1R3B	PPP1R3C	GSK3B	PHKA2
Stage IIA and -B (n=36)	16.2	19.7	16.7	16.9	18.3	21.1	17.1	19.1	11.6	17.6	19.4	17.5	16.9
	(14.9-17.0)	(15.9-20.9)	(14.8-17.7)	(15.4-17.9)	(17.5-19.2)	(18.9-22.9)	(16.3-17.7)	(17.3-20.1)	(0.0-15.3)	(15.1-19.1)	(15.0-21.4)	(16.2-18.1)	(15.3-17.8)
Stage IIIA-C (n=40)	16.3	19.0	16.1	16.7	18.2	20.6	17.0	19.0	10.6	16.9	18.1	17.3	16.6
	(15.3-17.2)	(16.1-21.7)	(14.5-17.5)	(15.7-19.2)	(17.6-19.1)	(19.0-23.1)	(16.2-17.9)	(17.9-20.2)	(0.0-15.7)	(15.2-20.4)	(15.6-21.1)	(16.7-18.0)	(15.8-18.3)
Stage IV (n=4)	16.0	18.8	15.5	16.6	18.4	20.3	16.6	18.6	10.5	15.8	17.3	17.4	16.3
	(15.6-16.6)	(17.5-19.5)	(15.0-16.2)	(16.1-16.9)	(18.0-19.0)	(19.7-21.3)	(16.4-16.8)	(18.5-18.8)	(0.0-11.1)	(15.4-17.3)	(16.7-18.2)	(16.9-18.0)	(16.2-16.5)
p - value[††]	0.54	0.14	**0.00**	0.36	0.13	0.15	0.20	0.22	0.18	**0.04**	**0.01**	0.31	**0.09**
**Extrascleral Extension (n=80)**	ATG7	BAP1	EPM2A	GBE1	GYG1	GYG2	INSR	IRS2	IRS4	PPP1R3B	PPP1R3C	GSK3B	PHKA2
No (n=71)	16.2	19.6	16.2	16.8	18.2	20.9	17.0	19.1	10.9	17.4	18.6	17.4	16.8
	(14.9-17.2)	(16.1-21.7)	(14.5-17.7)	(15.4-19.2)	(17.5-19.2)	(18.9-23.1)	(16.2-17.9)	(17.3-20.2)	(0.0-15.7)	(15.1-20.4)	(15.0-21.4)	(16.2-18.1)	(15.3-18.3)
Yes (n=9)	16.1	17.7	15.3	16.7	18.2	20.7	16.6	18.6	10.2	16.0	17.3	17.3	16.5
	(15.5-16.7)	(15.9-20.3)	(14.8-16.6)	(16.1-17.5)	(17.7-19.0)	(19.0-21.3)	(16.2-17.3)	(18.0-19.5)	(0.0-13.1)	(15.4-17.9)	(16.1-20.0)	(16.9-18.0)	(15.8-16.7)
p - value[†]	0.44	0.10	**0.02**	0.65	0.90	0.16	**0.04**	0.11	**0.04**	**0.01**	**0.02**	0.99	**0.01**
**BAP1 expression (n=80)**	ATG7	BAP1	EPM2A	GBE1	GYG1	GYG2	INSR	IRS2	IRS4	PPP1R3B	PPP1R3C	GSK3B	PHKA2
>Median (n=40)	16.4	20.1	16.6	17.2	18.4	21.3	17.1	19.2	13.2	18.0	19.5	17.6	17.1
	(15.8-17.2)	(19.5-21.7)	(15.1-17.7)	(15.4-19.2)	(17.7-19.2)	(20.2-23.1)	(16.3-17.9)	(18.5-20.2)	(0.0-15.3)	(15.4-20.4)	(16.7-21.4)	(16.2-18.1)	(16.1-18.3)
≤Median (n=40)	16.0	17.8	16.0	16.5	18.2	20.1	16.7	18.7	10.2	16.4	17.6	17.3	16.4
	(14.9-17.0)	(15.9-19.5)	(14.5-17.5)	(15.6-17.6)	(17.5-18.7)	(18.9-21.9)	(16.2-17.7)	(17.3-19.7)	(0.0-15.7)	(15.1-18.3)	(15.0-21.1)	(16.7-17.9)	(15.3-17.5)
p - value[†]	**0.00**	**0.00**	**0.00**	**0.00**	**0.00**	**0.00**	**0.00**	**0.00**	**0.00**	**0.00**	**0.00**	**0.00**	**0.00**
**Morphology (n=80)**	ATG7	BAP1	EPM2A	GBE1	GYG1	GYG2	INSR	IRS2	IRS4	PPP1R3B	PPP1R3C	GSK3B	PHKA2
Spindle (n=43)	16.3	19.8	16.5	16.9	18.3	21.3	17.1	19.1	11.2	17.7	19.4	17.5	17.1
	(14.9-17.2)	(16.1-21.7)	(15.0-17.5)	(15.4-19.2)	(17.5-19.2)	(19.4-23.1)	(16.3-17.9)	(17.9-20.2)	(0.0-15.7)	(15.4-20.4)	(16.6-21.3)	(16.2-18.1)	(16.1-18.3)
Mixed / Spindle (n=14)	16.3	18.8	15.8	16.5	18.2	20.8	17.0	19.1	10.6	17.2	18.0	17.3	16.6
	(15.8-16.6)	(15.9-21.0)	(14.8-17.7)	(15.9-17.7)	(17.6-18.6)	(19.0-21.8)	(16.4-17.3)	(18.3-20.2)	(0.0-14.6)	(15.7-20.0)	(16.1-20.2)	(16.7-18.0)	(16.3-17.9)
Mixed /Epithelioid (n=7)	16.0	17.4	15.7	16.2	18.2	20.1	16.6	18.6	10.5	16.1	17.9	17.3	16.4
	(15.8-17.0)	(16.3-19.9)	(14.5-17.6)	(15.7-17.9)	(18.0-18.7)	(19.5-21.0)	(16.2-17.1)	(18.1-19.2)	(0.0-15.3)	(15.2-18.5)	(17.0-19.9)	(17.2-17.8)	(16.0-16.6)
Epithelioid (n=16)	15.9	17.9	15.9	16.7	18.1	19.8	16.6	18.5	10.6	16.2	17.1	17.4	16.1
	(15.5-16.7)	(16.3-19.9)	(14.7-17.2)	(15.6-17.5)	(17.6-18.7)	(18.9-21.0)	(16.2-17.3)	(17.3-19.4)	(0.0-14.4)	(15.1-17.9)	(15.0-21.4)	(16.8-17.8)	(15.3-16.8)
p - value[††]	**0.01**	**0.00**	**0.00**	0.10	**0.06**	**0.00**	**0.00**	**0.00**	0.32	**0.00**	**0.00**	0.48	**0.00**
**Closed connective tissue loops (n=80)**	ATG7	BAP1	EPM2A	GBE1	GYG1	GYG2	INSR	IRS2	IRS4	PPP1R3B	PPP1R3C	GSK3B	PHKA2
Absent (n=38)	16.4	19.8	16.3	16.9	18.3	21.0	17.1	19.1	11.9	17.8	19.4	17.5	17.0
	(15.3-17.2)	(15.9-21.7)	(14.8-17.6)	(15.4-19.2)	(17.7-19.2)	(18.9-22.9)	(16.3-17.7)	(17.6-20.2)	(0.0-15.3)	(15.6-20.4)	(15.0-21.3)	(16.2-18.1)	(15.3-18.3)
Present (n=42)	16.1	18.8	16.1	16.7	18.2	20.6	16.8	18.9	10.4	16.6	18.1	17.3	16.6
	(14.9-17.0)	(16.1-20.8)	(14.5-17.7)	(15.6-17.9)	(17.5-19.1)	(19.0-23.1)	(16.2-17.9)	(17.3-20.2)	(0.0-15.7)	(15.1-18.9)	(15.9-21.4)	(16.7-18.0)	(15.5-18.0)
p - value[†]	**0.01**	**0.00**	0.34	0.23	**0.04**	**0.06**	**0.03**	0.22	**0.00**	**0.00**	**0.02**	0.17	**0.07**
**Mitotic count (n=80)**	ATG7	BAP1	EPM2A	GBE1	GYG1	GYG2	INSR	IRS2	IRS4	PPP1R3B	PPP1R3C	GSK3B	PHKA2
0-5 (n=63)	16.3	19.6	16.3	16.7	18.3	20.9	17.0	19.0	10.9	17.5	18.8	17.4	16.8
	(14.9-17.2)	(15.9-21.7)	(14.5-17.7)	(15.4-19.2)	(17.5-19.2)	(18.9-23.1)	(16.2-17.9)	(17.3-20.2)	(0.0-15.7)	(15.1-20.4)	(15.0-21.4)	(16.2-18.1)	(15.3-18.3)

>5-10 (n=11)	16.1 (15.6-16.4)	19.3 (17.5-20.3)	16.1 (15.0-17.2)	16.8 (16.1-17.7)	18.0 (17.6-18.5)	21.0 (19.0-22.6)	16.8 (16.4-17.3)	19.1 (18.5-20.2)	10.9 (8.1-14.3)	16.7 (15.4-18.8)	18.0 (16.9-20.3)	17.6 (16.9-17.9)	16.5 (16.1-17.9)
>11 (n=6)	16.1 (15.6-16.5)	18.9 (16.3-19.8)	16.0 (14.6-16.5)	16.6 (15.9-17.9)	18.3 (17.9-18.7)	20.0 (19.4-20.5)	16.7 (16.3-17.3)	18.5 (18.1-19.4)	10.7 (0.0-13.0)	16.1 (15.6-17.6)	17.4 (15.6-18.9)	17.3 (16.9-17.6)	16.5 (16.0-17.2)
p - value††	0.11	0.34	0.23	0.82	**0.05**	**0.03**	0.42	0.24	0.54	**0.06**	**0.04**	0.48	0.40
**Tumor infiltrating lymphocytes density (n=80)**	**ATG7**	**BAP1**	**EPM2A**	**GBE1**	**GYG1**	**GYG2**	**INSR**	**IRS2**	**IRS4**	**PPP1R3B**	**PPP1R3C**	**GSK3B**	**PHKA2**
Mild (n=71)	16.3 (14.9-17.2)	19.6 (16.1-21.7)	16.2 (14.5-17.7)	16.8 (15.7-19.2)	18.3 (17.5-19.2)	20.9 (18.9-23.1)	17.0 (16.2-17.9)	19.1 (17.3-20.2)	10.9 (0.0-15.7)	17.4 (15.2-20.4)	18.7 (15.6-21.4)	17.5 (16.7-18.1)	16.8 (15.8-18.3)
Moderate (n=7)	16.0 (15.5-16.7)	17.5 (16.3-19.9)	15.9 (14.7-16.6)	16.4 (15.4-17.5)	18.1 (17.7-18.7)	19.8 (19.0-22.2)	16.6 (16.2-17.5)	18.6 (17.6-19.1)	9.2 (0.0-13.1)	16.1 (15.1-17.9)	17.3 (15.0-18,4)	17.2 (16.2-17.8)	16.1 (15.3-16.7)
Heavy (n=2)	16.1 (15.8-16.4)	17.5 (15.9-19.0)	15.4 (14.8-15.9)	15.9 (15.6-16.2)	18.0 (17.9-18.2)	19.9 (19.4-20.4)	16.7 (16.5-16.8)	18.7 (18.5-18.8)	10.1 (9.4-10.9)	16.0 (16.0-16.1)	16.0 (15.9-16.1)	16.9 (16.8-17.1)	16.0 (15.8-16.3)
p - value††	0.22	**0.06**	**0.05**	**0.03**	0.17	0.11	0.24	**0.03**	**0.07**	**0.02**	**0.00**	**0.06**	**0.00**
**Tumor infiltrating macrophages density (n=80)**	**ATG7**	**BAP1**	**EPM2A**	**GBE1**	**GYG1**	**GYG2**	**INSR**	**IRS2**	**IRS4**	**PPP1R3B**	**PPP1R3C**	**GSK3B**	**PHKA2**
Mild (n=40)	16.1 (15.3-17.1)	19.6 (16.1-21.3)	16.4 (15.0-17.7)	16.9 (15.9-19.2)	18.3 (17.6-19.2)	21.0 (19.0-22.7)	17.1 (16.4-17.7)	19.1 (17.9-20.1)	11.2 (0.0-15.7)	17.6 (15.4-20.4)	19.4 (16.6-21.4)	17.6 (16.7-18.1)	16.9 (16.1-18.3)
Moderate (n=29)	16.2 (14.9-17.1)	19.0 (15.9-21.7)	16.0 (14.6-17.4)	16.6 (15.9-17.7)	18.2 (17.5-19.1)	20.6 (18.9-23.1)	16.8 (16.2-17.9)	18.9 (17.6-20.2)	10.6 (0.0-14.6)	16.8 (15.1-18.9)	18.0 (15.0-21.1)	17.3 (16.8-17.9)	16.6 (15.3-18.0)
Heavy (n=11)	16.3 (15.8-17.2)	19.0 (16.6-21.0)	15.9 (14.5-17.0)	16.6 (15.4-17.1)	18.2 (17.9-18.4)	20.7 (19.4-22.9)	16.6 (16.2-17.5)	18.8 (17.3-20.0)	10.5 (8.2-14.6)	16.8 (15.2-19.1)	17.8 (15.9-19.6)	17.3 (16.2-18.1)	16.5 (15.8-17.5)
p - value††	0.29	0.39	**0.00**	**0.00**	**0.08**	0.39	0.12	0.43	**<0.05**	**0.03**	**0.00**	**0.02**	0.49
**Pigmentation (n=80)**	**ATG7**	**BAP1**	**EPM2A**	**GBE1**	**GYG1**	**GYG2**	**INSR**	**IRS2**	**IRS4**	**PPP1R3B**	**PPP1R3C**	**GSK3B**	**PHKA2**
Mild (n=39)	16.1 (14.9-17.1)	19.6 (16.1-21.3)	16.5 (15.3-17.7)	17.0 (16.1-19.2)	18.3 (17.5-19.2)	20.9 (18.9-22.7)	17.1 (16.3-17.7)	19.1 (17.9-20.1)	11.4 (0.0-15.7)	17.6 (15.6-20.4)	19.3 (15.9-21.4)	17.6 (16.7-18.1)	17.0 (16.1-18.3)
Moderate (n=27)	16.2 (15.5-17.2)	19.0 (16.2-21.7)	15.9 (14.6-16.9)	16.6 (15.6-17.6)	18.2 (17.7-19.0)	20.7 (19.0-23.1)	16.8 (16.2-17.9)	18.8 (17.3-20.2)	10.3 (0.0-14.6)	16.6 (15.1-18.9)	17.8 (15.0-20.8)	17.3 (16.8-18.0)	16.6 (15.3-18.0)
Heavy (n=14)	16.4 (15.7-17.0)	19.2 (15.9-20.9)	15.8 (14.5-17.2)	16.5 (15.4-17.2)	18.2 (17.9-18.9)	20.6 (19.4-22.9)	16.6 (16.2-17.7)	19.1 (18.3-20.0)	10.1 (0.0-13.5)	16.9 (15.2-19.1)	18.5 (15.6-20.1)	17.3 (16.2-18.1)	16.5 (15.8-17.2)
p - value††	0.34	0.67	**0.00**	**0.00**	0.43	0.72	0.11	0.28	**0.00**	**0.01**	**0.00**	**0.04**	**0.08**
**Necrosis (n=80)**	**ATG7**	**BAP1**	**EPM2A**	**GBE1**	**GYG1**	**GYG2**	**INSR**	**IRS2**	**IRS4**	**PPP1R3B**	**PPP1R3C**	**GSK3B**	**PHKA2**
Absent (n=63)	16.2 (15.3-17.2)	19.6 (16.1-21.7)	16.2 (14.5-17.7)	16.8 (15.4-17.9)	18.3 (17.5-19.2)	20.9 (19.0-23.1)	17.0 (16.2-17.9)	19.1 (17.6-20.2)	10.9 (0.0-15.7)	17.3 (15.1-20.4)	18.8 (15.0-21.4)	17.4 (16.2-18.1)	16.8 (15.3-18.0)
Present (n=17)	16.2 (14.9-16.8)	19.3 (15.9-21.0)	15.9 (14.6-17.2)	16.7 (16.1-19.2)	18.2 (17.6-19.0)	20.7 (18.9-22.9)	16.8 (16.2-17.6)	18.7 (17.3-20.2)	10.9 (0.0-14.6)	16.8 (15.4-18.6)	17.7 (15.9-20.6)	17.4 (16.9-18.0)	16.5 (16.0-18.3)
p - value†	0.72	0.69	**0.09**	0.60	0.21	0.27	0.45	0.40	0.12	0.51	**0.00**	0.86	0.14
**Body-Mass-Index (n=53)**	**ATG7**	**BAP1**	**EPM2A**	**GBE1**	**GYG1**	**GYG2**	**INSR**	**IRS2**	**IRS4**	**PPP1R3B**	**PPP1R3C**	**GSK3B**	**PHKA2**
<25.0 (n=17)	16.2 (14.9-16.9)	19.6 (16.2-20.9)	16.4 (15.3-17.4)	16.9 (15.6-17.7)	18.2 (17.6-18.9)	20.9 (19.4-23.1)	17.2 (16.5-17.9)	19.1 (17.3-20.2)	11.4 (0.0-14.9)	17.7 (15.2-19.1)	19.0 (15.9-21.4)	17.5 (16.8-18.1)	16.9 (15.8-18.0)

≥25.0 (n=36)	16.2<br>(15.3-17.2)	19.4<br>(16.1-21.7)	16.0<br>(14.5-17.6)	16.7<br>(15.4-17.9)	18.3<br>(17.7-19.1)	20.8<br>(18.9-22.7)	16.9<br>(16.2-17.7)	18.9<br>(17.9-20.2)	10.8<br>(0.0-15.3)	17.1<br>(15.3-20.4)	18.3<br>(15.6-20.4)	17.4<br>(16.2-18.0)	16.5<br>(15.8-17.6)
p - value†	0.82	0.47	**0.08**	0.32	0.20	0.27	0.12	**<0.05**	0.47	0.65	0.80	0.67	0.14
Progression (n=80)	ATG7	BAP1	EPM2A	GBE1	GYG1	GYG2	INSR	IRS2	IRS4	PPP1R3B	PPP1R3C	GSK3B	PHKA2
Censored (n=50)	16.3<br>(15.5-17.2)	19.8<br>(16.1-21.7)	16.4<br>(14.6-17.7)	16.9<br>(15.4-19.2)	18.3<br>(17.5-19.2)	21.1<br>(19.4-23.1)	17.1<br>(16.3-17.9)	19.1<br>(17.3-20.2)	11.3<br>(0.0-15.7)	17.6<br>(15.6-20.4)	19.1<br>(15.0-21.4)	17.5<br>(16.2-18.1)	17.0<br>(15.3-18.3)
Progression (n=30)	16.0<br>(14.9-17.0)	18.0<br>(15.9-20.6)	15.6<br>(14.5-17.4)	16.6<br>(15.7-17.7)	18.1<br>(17.7-19.0)	20.2<br>(18.9-22.6)	16.7<br>(16.2-17.4)	18.7<br>(17.9-20.1)	10.5<br>(0.0-15.0)	16.4<br>(15.1-18.8)	17.3<br>(15.6-20.4)	17.4<br>(16.8-18.0)	16.5<br>(15.5-17.8)
p - value†	**0.01**	**0.00**	**0.00**	**0.02**	**0.01**	**0.00**	**0.00**	**0.00**	**0.09**	**0.00**	**0.00**	0.55	**0.00**
Metastases (n=80)	ATG7	BAP1	EPM2A	GBE1	GYG1	GYG2	INSR	IRS2	IRS4	PPP1R3B	PPP1R3C	GSK3B	PHKA2
No (n=54)	16.3<br>(15.5-17.2)	19.8<br>(16.1-21.7)	16.4<br>(14.6-17.7)	16.9<br>(15.4-19.2)	18.3<br>(17.5-19.2)	21.1<br>(19.0-23.1)	17.1<br>(16.2-17.9)	19.1<br>(17.3-20.2)	11.7<br>(0.0-15.7)	17.6<br>(15.6-20.4)	19.2<br>(15.0-21.4)	17.5<br>(16.2-18.1)	17.0<br>(15.3-18.3)
Yes (n=26)	16.0<br>(14.9-17.0)	17.9<br>(15.9-20.3)	15.6<br>(14.5-16.9)	16.5<br>(15.7-17.7)	18.1<br>(17.7-19.0)	19.9<br>(18.9-22.6)	16.6<br>(16.2-17.7)	18.7<br>(17.9-20.1)	10.5<br>(0.0-14.3)	16.3<br>(15.1-18,8)	17.3<br>(15.6-20.3)	17.3<br>(16.8-18.0)	16.3<br>(15.5-17.6)
p - value†	**0.01**	**0.00**	**0.00**	**0.00**	**0.02**	**0.00**	**0.00**	**0.00**	**0.02**	**0.00**	**0.00**	0.15	**0.00**
Disease specific survival (n=78)	ATG7	BAP1	EPM2A	GBE1	GYG1	GYG2	INSR	IRS2	IRS4	PPP1R3B	PPP1R3C	GSK3B	PHKA2
Alive or dead due to other causes (n=58)	16.3<br>(15.3-17.2)	19.8<br>(16.1-21.7)	16.4<br>(14.6-17.7)	16.9<br>(15.4-19.2)	18.3<br>(17.5-19.2)	21.2<br>(19.0-23.1)	17.1<br>(16.3-17.9)	19.1<br>(17.3-20.2)	11.7<br>(0.0-15.7)	17.6<br>(15.1-20.4)	19.2<br>(15.0-21.4)	17.5<br>(16.2-18.1)	17.0<br>(15.3-18.3)
Dead due to UM-metastases (n=20)	15.9<br>(14.9-17.0)	17.9<br>(15.9-20.2)	15.6<br>(14.5-16.8)	16.5<br>(15.7-16.9)	18.2<br>(17.9-19.0)	19.9<br>(18.9-21.5)	16.6<br>(16.2-17.7)	18.7<br>(18.0-19.4)	10.5<br>(0.0-11.5)	16.3<br>(15.2-17.3)	17.2<br>(15.6-19.5)	17.3<br>(16.9-18.0)	16.3<br>(15.8-16.9)
p - value†	**0.03**	**0.00**	**0.00**	**0.00**	**0.08**	**0.00**	**0.00**	**0.00**	**0.03**	**0.00**	**0.00**	**0.04**	**0.00**

*References for gene function are listed in Supplementary Table 1. Genes with a differential expression are highlighted in color. n: number. † Mann-Whitney U test · †† Kruskal-Wallis test

Table S3. Genes with a higher expression level in the monosomy-3 tumors regardless of the gene copy number (n = 9).

Supplementary Table S3

Genes with a higher expression level in the monosomy-3 tumors regardless of the gene copy number (n=9) · Median expression (range)

Function*	#1,2. Dual regulators of glycogen synthesis		#3-9. Negative regulators of glycogen synthesis						
Gene symbol	PGM2	PGM5	CALM1	CALM2	G6PC3	GFPT1	PCK1	PPP1R14C	PYGM
Locus	4p14	9q21.11	14q32.11	2p21	17q21.31	2p13.3	20q13.31	6q25.1	11q13.1
Expression (n=80)	17.4<br>(15.0-19.5)	12.3<br>(8.3-15.5)	20.0<br>(18.9-20.9)	19.8<br>(18.2-21.1)	20.4<br>(18.5-22.3)	17.0<br>(15.5-18.4)	8.4<br>(0.0-12.8)	10.9<br>(0.0-16.4)	13.2<br>(10.2-16.2)
Age (n=80)	PGM2	PGM5	CALM1	CALM2	G6PC3	GFPT1	PCK1	PPP1R14C	PYGM
≤Median (n=40)	17.5<br>(16.0-19.5)	12.5<br>(10.0-15.0)	20.0<br>(19.1-20.6)	19.7<br>(18.5-20.7)	20.4<br>(18.5-22.3)	17.0<br>(15.5-18.4)	8.3<br>(0.0-12.8)	10.4<br>(0.0-16.4)	13.0<br>(10.3-16.2)
>Median (n=40)	17.3<br>(15.0-18.9)	12.2<br>(8.3-15.5)	19.9<br>(18.9-20.9)	20.0<br>(18.2-21.1)	20.4<br>(18.8-22.0)	17.0<br>(15.5-18.4)	8.7<br>(0.0-12.2)	11.0<br>(0.0-14.8)	13.3<br>(10.2-16.0)

	PGM2	PGM5	CALM1	CALM2	G6PC3	GFPT1	PCK1	PPP1R14C	PYGM
p - value[†]	0.83	0.20	0.95	0.55	0.43	0.60	0.82	0.56	0.28
Gender (n=80)	PGM2	PGM5	CALM1	CALM2	G6PC3	GFPT1	PCK1	PPP1R14C	PYGM
Female (n=35)	17.5 (15.0-19.5)	12.2 (8.3-14.2)	19.9 (18.9-20.6)	19.8 (18.5-20.7)	20.4 (18.7-21.7)	16.9 (15.5-18.4)	8.9 (0.0-12.8)	11.1 (0.0-16.4)	13.4 (11.0-16.2)
Male (n=45)	17.4 (15.8-18.9)	12.3 (10.0-15.5)	20.0 (19.1-20.9)	19.9 (18.2-21.1)	20.5 (18.5-22.3)	17.0 (15.8-18.4)	8.3 (0.0-11.7)	9.9 (0.0-14.4)	13.0 (10.2-15.7)
p - value[†]	0.62	0.72	0.46	0.74	0.74	0.93	0.48	0.57	0.17
Eye color (n=30)	PGM2	PGM5	CALM1	CALM2	G6PC3	GFPT1	PCK1	PPP1R14C	PYGM
Brown (n=15)	17.1 (15.8-19.5)	12.0 (10.0-15.0)	20.0 (19.4-20.5)	19.6 (18.2-20.7)	20.7 (19.9-22.3)	16.9 (15.5-18.0)	7.3 (0.0-11.7)	9.9 (0.0-14.4)	12.5 (11.5-15.7)
Green (n=6)	17.9 (17.5-18.9)	12.0 (8.3-14.3)	19.8 (19.4-20.0)	19.8 (19.0-21.1)	20.4 (18.8-21.4)	16.9 (16.3-18.4)	8.8 (7.6-10.1)	12.0 (0.0-14.8)	13.7 (12.2-14.5)
Blue (n=9)	17.2 (15.0-18.4)	12.4 (11.0-13.7)	19.9 (18.9-20.6)	19.7 (18.7-20.5)	20.6 (18.9-21.7)	16.6 (15.7-17.8)	9.0 (0.0-12.2)	9.9 (0.0-16.4)	13.9 (11.0-16.0)
p - value[††]	0.13	0.85	0.58	0.96	0.61	0.40	0.50	0.91	0.42
Largest Basal Diameter (n=74)	PGM2	PGM5	CALM1	CALM2	G6PC3	GFPT1	PCK1	PPP1R14C	PYGM
≤13 mm (n=12)	16.9 (16.0-18.9)	11.9 (8.3-13.9)	19.9 (18.9-20.4)	19.5 (18.5-21.1)	20.6 (18.7-21.7)	16.6 (15.7-18.4)	7.0 (0.0-10.1)	9.0 (0.0-11.9)	12.8 (10.6-15.4)
>13 mm (n=62)	17.5 (15.0-19.5)	12.3 (10.5-15.5)	20.0 (18.9-20.9)	20.0 (18.2-20.7)	20.4 (18.5-22.3)	17.0 (15.5-18.4)	8.9 (0.0-12.8)	11.3 (0.0-14.8)	13.2 (10.2-16.2)
p - value[†]	0.25	0.19	0.88	0.11	0.64	**0.06**	**0.04**	**0.03**	0.45
Tumor thickness (n=76)	PGM2	PGM5	CALM1	CALM2	G6PC3	GFPT1	PCK1	PPP1R14C	PYGM
< Median (n=37)	17.4 (15.9-18.9)	12.5 (10.3-15.5)	19.9 (18.9-20.9)	19.9 (18.5-20.6)	20.4 (18.7-22.3)	16.9 (15.5-18.3)	8.8 (0.0-12.2)	10.8 (0.0-14.7)	13.1 (10.2-16.0)
≥ Median (n=39)	17.4 (15.0-19.5)	12.0 (8.3-14.3)	20.0 (18.9-20.8)	19.6 (18.2-21.1)	20.5 (18.5-22.0)	17.0 (15.5-18.4)	8.3 (0.0-12.8)	11.3 (0.0-14.8)	13.2 (10.3-16.2)
p - value[†]	0.96	0.20	0.43	0.26	0.39	0.67	0.98	0.96	0.93
AJCC Stage, clinical (n=80)	PGM2	PGM5	CALM1	CALM2	G6PC3	GFPT1	PCK1	PPP1R14C	PYGM
Stage IIA and -B (n=36)	17.2 (15.0-18.9)	12.0 (10.3-13.9)	19.9 (18.9-20.8)	19.7 (18.5-20.6)	20.2 (18.7-21.9)	16.8 (15.5-18.0)	7.6 (0.0-9.9)	9.6 (0.0-13.7)	13.0 (10.2-15.4)
Stage IIIA-C (n=40)	17.5 (15.8-19.5)	12.4 (8.3-15.5)	20.0 (19.1-20.9)	19.8 (18.2-21.1)	20.5 (18.5-22.3)	17.0 (15.5-18.4)	9.0 (0.0-12.8)	11.6 (0.0-14.8)	13.4 (10.3-16.2)
Stage IV (n=4)	18.0 (17.5-18.4)	13.0 (12.4-13.4)	20.2 (20.1-20.6)	20.3 (20.1-20.5)	20.7 (19.8-21.5)	17.5 (16.6-17.8)	9.9 (6.1-12.2)	14.1 (9.6-16.4)	13.7 (12.8-16.0)

p - value††	**0.06**	0.12	**0.06**	**0.06**	0.24	0.26	**0.01**	**<0.01**	**0.08**
Extrascleral Extension (n=80)	PGM2	PGM5	CALM1	CALM2	G6PC3	GFPT1	PCK1	PPP1R14C	PYGM
No (n=71)	17.4 (15.0-19.5)	12.3 (8.3-15.5)	19.9 (18.9-20.9)	19.8 (18.2-20.7)	20.4 (18.5-22.3)	16.9 (15.5-18.4)	8.3 (0.0-12.8)	9.9 (0.0-16.4)	13.1 (10.2-16.2)
Yes (n=9)	17.5 (16.4-18.9)	12.4 (10.9-14.4)	20.1 (19.6-20.4)	20.2 (19.6-21.1)	20.7 (19.1-21.9)	17.6 (16.6-18.4)	9.2 (0.0-12.2)	11.9 (8.7-14.8)	13.9 (11.5-16.0)
p - value†	0.36	0.65	0.25	**0.06**	0.30	**0.04**	**0.06**	**<0.05**	0.16
BAP1 expression (n=80)	PGM2	PGM5	CALM1	CALM2	G6PC3	GFPT1	PCK1	PPP1R14C	PYGM
>Median (n=40)	17.1 (15.0-18.3)	12.0 (8.3-14.2)	19.7 (18.9-20.4)	19.5 (18.2-20.3)	20.1 (18.5-21.7)	16.6 (15.5-17.5)	7.1 (0.0-10.3)	8.0 (0.0-14.4)	12.5 (10.2-15.7)
≤Median (n=40)	17.9 (16.5-19.5)	12.6 (10.5-15.5)	20.2 (19.2-20.9)	20.1 (19.1-21.1)	20.6 (18.9-22.3)	17.4 (16.5-18.4)	9.3 (0.0-12.8)	12.3 (0.0-16.4)	13.8 (10.3-16.2)
p - value†	**<0.01**	**<0.01**	**<0.01**	**<0.01**	**<0.01**	**<0.01**	**<0.01**	**<0.01**	**<0.01**
Morphology (n=80)	PGM2	PGM5	CALM1	CALM2	G6PC3	GFPT1	PCK1	PPP1R14C	PYGM
Spindle (n=43)	17.3 (15.0-18.7)	12.0 (8.3-14.3)	19.8 (18.9-20.6)	19.6 (18.2-20.5)	20.1 (18.5-21.7)	16.8 (15.5-17.9)	7.5 (0.0-12.8)	8.9 (0.0-16.4)	12.7 (10.3-16.0)
Mixed / Spindle (n=14)	17.2 (16.1-18.2)	12.7 (10.7-15.5)	20.0 (19.4-20.9)	19.6 (18.7-20.2)	20.6 (18.8-21.9)	16.8 (15.8-17.7)	8.9 (0.0-10.3)	11.7 (0.0-14.4)	13.5 (11.4-16.2)
Mixed /Epithelioid (n=7)	17.6 (16.5-18.2)	12.9 (11.9-14.4)	20.1 (19.9-20.4)	20.1 (19.7-20.2)	20.4 (19.4-22.3)	17.4 (16.9-18.3)	9.5 (0.0-11.7)	12.2 (9.2-13.2)	14.0 (10.2-15.6)
Epithelioid (n=16)	17.9 (16.5-19.5)	12.5 (11.6-14.2)	20.2 (19.4-20.8)	20.1 (19.6-21.1)	20.6 (19.1-21.6)	17.9 (16.6-18.4)	9.3 (0.0-11.4)	12.1 (9.7-14.8)	13.6 (11.2-15.5)
p - value††	**0.06**	**0.02**	**<0.01**	**<0.01**	0.19	**<0.01**	**0.01**	**<0.01**	**0.05**
Closed connective tissue loops (n=80)	PGM2	PGM5	CALM1	CALM2	G6PC3	GFPT1	PCK1	PPP1R14C	PYGM
Absent (n=38)	17.1 (15.0-19.5)	12.0 (10.0-15.0)	19.8 (18.9-20.8)	19.6 (18.2-20.7)	20.2 (18.5-21.9)	16.8 (15.5-18.0)	7.5 (0.0-9.9)	8.9 (0.0-14.4)	13.0 (10.2-15.4)
Present (n=42)	17.7 (16.0-18.9)	12.5 (8.3-15.5)	20.0 (19.2-20.9)	20.0 (19.0-21.1)	20.5 (18.9-22.3)	17.1 (15.5-18.4)	9.1 (0.0-12.8)	11.8 (0.0-16.4)	13.5 (10.3-16.2)
p - value†	**<0.01**	0.23	**0.01**	**<0.01**	0.35	**<0.01**	**<0.01**	**<0.01**	**0.02**
Mitotic count (n=80)	PGM2	PGM5	CALM1	CALM2	G6PC3	GFPT1	PCK1	PPP1R14C	PYGM
0-5 (n=63)	17.3 (15.0-18.9)	12.2 (8.3-15.0)	19.9 (18.9-20.8)	19.7 (18.2-21.1)	20.4 (18.5-22.3)	16.9 (15.5-18.4)	8.1 (0.0-12.8)	9.8 (0.0-14.8)	13.0 (10.2-16.2)
>5-10 (n=11)	17.6 (16.8-18.4)	12.4 (11.1-15.5)	20.1 (19.4-20.9)	20.0 (19.2-20.5)	20.0 (19.1-21.7)	16.9 (15.8-18.4)	9.3 (6.1-12.2)	11.9 (0.0-16.4)	13.9 (11.4-16.0)

>11 (n=6)	17.7 (16.9-19.5)	12.6 (12.0-13.1)	20.2 (19.7-20.4)	20.1 (19.9-20.7)	20.6 (19.2-22.0)	17.5 (16.6-18.0)	9.6 (7.2-9.9)	11.9 (7.9-13.3)	13.0 (11.5-13.7)
p - value††	0.14	0.66	0.18	0.10	0.83	0.25	**0.01**	**0.05**	**0.09**
Tumor infiltrating lymphocytes density (n=80)	PGM2	PGM5	CALM1	CALM2	G6PC3	GFPT1	PCK1	PPP1R14C	PYGM
Mild (n=71)	17.5 (15.0-19.5)	12.2 (8.3-15.5)	19.9 (18.9-20.9)	19.8 (18.2-21.1)	20.3 (18.5-22.3)	16.9 (15.5-18.4)	8.3 (0.0-12.8)	9.9 (0.0-16.4)	13.0 (10.2-16.2)
Moderate (n=7)	17.4 (16.0-17.9)	13.5 (12.5-14.2)	20.3 (19.7-20.5)	20.2 (18.5-20.6)	20.7 (19.1-21.3)	17.8 (16.6-18.0)	9.1 (0.0-11.4)	13.2 (9.8-14.2)	13.9 (13.2-15.5)
Heavy (n=2)	17.1 (16.9-17.3)	12.2 (11.8-12.7)	20.3 (20.1-20.6)	19.7 (19.6-19.8)	21.2 (20.6-21.9)	17.6 (17.4-17.7)	9.3 (8.6-9.9)	11.9 (11.6-12.2)	13.7 (13.5-13.9)
p - value††	0.77	**0.04**	**0.02**	0.32	0.26	**0.05**	0.19	**0.05**	**0.03**
Tumor infiltrating macrophages density (n=80)	PGM2	PGM5	CALM1	CALM2	G6PC3	GFPT1	PCK1	PPP1R14C	PYGM
Mild (n=40)	17.5 (16.1-18.7)	12.3 (10.7-15.5)	19.8 (18.9-20.9)	19.8 (18.8-20.5)	19.9 (18.5-21.7)	17.0 (15.5-18.4)	7.4 (0.0-12.8)	9.6 (0.0-16.4)	12.8 (10.2-16.2)
Moderate (n=29)	17.3 (15.8-19.5)	12.3 (8.3-14.2)	20.0 (19.5-20.5)	19.9 (18.2-21.1)	20.7 (19.1-22.0)	17.0 (15.5-18.4)	9.0 (0.0-11.4)	11.6 (0.0-14.8)	13.4 (10.6-15.7)
Heavy (n=11)	16.7 (15.0-18.9)	11.9 (10.0-15.0)	20.2 (18.9-20.8)	19.7 (18.5-20.4)	20.8 (19.9-22.3)	17.2 (15.9-18.0)	9.1 (0.0-11.7)	9.8 (0.0-12.8)	13.5 (11.4-15.6)
p - value††	0.30	0.90	**0.06**	0.56	**<0.01**	0.91	0.10	0.20	**0.05**
Pigmentation (n=80)	PGM2	PGM5	CALM1	CALM2	G6PC3	GFPT1	PCK1	PPP1R14C	PYGM
Mild (n=39)	17.5 (16.1-18.7)	12.3 (10.5-15.5)	19.9 (18.9-20.9)	19.9 (18.8-20.6)	19.9 (18.5-21.7)	17.0 (15.7-18.4)	7.5 (0.0-12.8)	9.9 (0.0-16.4)	12.9 (10.2-16.2)
Moderate (n=27)	17.2 (15.8-18.9)	12.7 (10.0-14.4)	20.1 (19.4-20.6)	19.9 (18.2-21.1)	20.7 (19.8-22.0)	17.1 (15.5-18.4)	8.9 (0.0-12.2)	11.6 (0.0-14.7)	13.4 (11.4-16.0)
Heavy (n=14)	17.0 (15.0-19.5)	12.0 (8.3-15.0)	20.0 (18.9-20.8)	19.6 (18.5-20.7)	20.9 (19.9-22.3	17.1 (16.2-18.0)	9.3 (0.0-11.7)	11.4 (0.0-14.8)	13.7 (10.6-15.6)
p - value††	0.30	0.64	0.15	0.50	**<0.01**	0.99	**0.07**	0.56	**0.06**
Necrosis (n=80)	PGM2	PGM5	CALM1	CALM2	G6PC3	GFPT1	PCK1	PPP1R14C	PYGM
Absent (n=63)	17.5 (15.8-19.5)	12.4 (10.0-15.5)	20.0 (18.9-20.9)	19.8 (18.2-21.1)	20.3 (18.7-22.3)	16.9 (15.5-18.4)	8.3 (0.0-12.8)	9.9 (0.0-16.4)	13.0 (10.2-16.2)
Present (n=17)	17.3 (15.0-18.9)	12.2 (8.3-14.2)	20.0 (18.9-20.7)	20.0 (18.7-20.6)	20.5 (18.5-22.0)	17.1 (16.0-18.0)	9.2 (0.0-11.4)	11.6 (0.0-14.8)	13.5 (11.4-15.5)
p - value†	0.90	0.37	0.84	0.37	0.18	0.37	0.14	0.34	0.34
Body-Mass-Index	PGM2	PGM5	CALM1	CALM2	G6PC3	GFPT1	PCK1	PPP1R14C	PYGM

(n=53)									
<25.0 (n=17)	17.2	12.2	19.9	19.7	20.5	16.8	8.4	11.9	13.4
	(15.0-18.9)	(8.3-13.0)	(18.9-20.7)	(19.0-20.5)	(18.9-21.7)	(15.5-18.0)	(0.0-10.3)	(0.0-14.8)	(11.0-15.7)
≥25.0 (n=36)	17.5	12.4	20.0	19.8	20.6	17.0	9.0	11.0	13.1
	(15.8-19.5)	(10.0-15.5)	(19.1-20.9)	(18.2-21.1)	(18.7-22.3)	(15.7-18.4)	(0.0-12.8)	(0.0-16.4)	(10.2-16.2)
p - value†	0.81	**0.07**	0.36	0.50	0.99	0.11	0.15	0.99	0.95
Progression (n=80)	PGM2	PGM5	CALM1	CALM2	G6PC3	GFPT1	PCK1	PPP1R14C	PYGM
Censored (n=50)	17.2	12.3	19.9	19.6	20.4	16.8	7.3	9.5	12.9
	(15.0-18.9	(8.3-14.4)	(18.9-20.7)	(18.2-20.5)	(18.5-22.0)	(15.5-18.3)	(0.0-12.8)	(0.0-16.4)	(10.2-15.7)
Progression (n=30)	17.8	12.3	20.1	20.1	20.4	17.4	9.3	12.1	13.4
	(16.5-19.5)	(10.5-15.5)	(19.1-20.9)	(19.2-21.1)	(18.7-22.3)	(16.3-18.4)	(0.0-12.2)	(0.0-14.8)	(11.0-16.2)
p - value†	**<0.01**	0.98	**0.02**	**<0.01**	0.52	**<0.01**	**<0.01**	**<0.01**	**0.04**
Metastases (n=80)	PGM2	PGM5	CALM1	CALM2	G6PC3	GFPT1	PCK1	PPP1R14C	PYGM
No (n=54)	17.2	12.1	19.8	19.6	20.2	16.8	7.5	9.1	12.8
	(15.0-18.9)	(8.3-15.5)	(18.9-20.9)	(18.2-20.5)	(18.5-22.0)	(15.5-18.0)	(0.0-12.8)	(0.0-14.8)	(10.2-15.7)
Yes (n=26)	17.9	12.6	20.2	20.2	20.6	17.5	9.3	12.3	13.5
	(15.9-19.5)	(10.5-15.0)	(19.4-20.8)	(19.2-21.1)	(19.1-22.3)	(16.2-18.4)	(0.0-12.2)	(8.0-16.4)	(12.7-16.2)
p - value†	**<0.01**	0.12	**<0.01**	**<0.01**	**0.05**	**<0.01**	**<0.01**	**<0.01**	**0.01**
Disease specific survival (n=78)	PGM2	PGM5	CALM1	CALM2	G6PC3	GFPT1	PCK1	PPP1R14C	PYGM
Alive or dead due to other causes (n=58)	17.3	12.2	19.8	19.7	20.1	16.8	7.6	9.2	12.9
	(15.0-18.9)	(8.3-15.5)	(18.9-20.9)	(18.2-20.6)	(18.5-22.0)	(15.5-18.0)	(0.0-12.8)	(0.0-14.8)	(10.2-16.0)
Dead due to UM-metastases (n=20)	17.9	12.8	20.1	20.1	20.6	17.6	9.4	12.3	13.6
	(15.9-19.5)	(10.5-15.0)	(19.7-20.8)	(19.5-21.1)	(19.2-22.3)	(16.2-18.4)	(0.0-11.7)	(9.6-16.4)	(12.7-16.2)
p - value†	**0.02**	**0.09**	**<0.01**	**<0.01**	**0.03**	**<0.01**	**<0.01**	**<0.01**	**0.02**

*References for gene function are listed in Supplementary Table 1. Genes with a differential expression are highlighted in color. n: number.
† Mann-Whitney U test · †† Kruskal-Wallis test

Table S4. Validation of the differentially expressed genes in the monosomy-3 tumors of the TCGA study in an independent UM cohort.

Supplementary Table S4. Validation of the differentially expressed genes in the monosomy-3 tumors of the TCGA study in an independent UM cohort (GEO accession: GSE44295)

Symbol	Locus	Probe ID	Mean ± SD	Median expression (range)									
				Metastases					Gender				
			(n=57)	No (n=33)	Yes (n=24)	Fold change* in metastatic patients	$p^{†}$	$p^{††}$	Female (n=25)	Male (n=32)	Fold change * in males	$p^{†}$	$p^{††}$

1. De novo glycogen synthesis**													
GBE1	3p12.2	ILMN_ 1789702	65.5 ± 86.9	42.2 (4.7_585.5)	29.0 (0.8_171.5)	-1.46	0.09	1.00	37.7 (0.8_210.2)	43.2 (2.9_585.5)	1.15	0.71	1.00
GYG1	3q24	ILMN_ 2230862	235.3 ± 225.3	132.0 (9.9_887.9)	105.7 (31.8_755.8)	-1.25	0.85	1.00	110.8 (31.8_773.2)	105.7 (9.9_887.9)	-1.05	0.47	1.00
**GYG2**	Xp22.33	ILMN_ 2245686	1.2 ± 8.5	-0.5 (-5.8_28.1)	-0.7 (-7.3_43.0)	n.a.	0.38	1.00	-0.9 (-7.3_28.1)	-0.3 (-5.8_43.0)	n.a.	0.36	1.00
		ILMN_ 2319424	1065.9 ± 754.1	1091.4 (273.9_5024.6)	660.4 (181.8_1602.9)	-1.65	**0.00**	**0.04**	1252.1 (655.4_5024.6)	631.9 (181.8_2439.0)	-1.98	**0.00**	**0.01**
		ILMN_ 1684017	117.4 ± 99.7	129.5 (-4.5_625.5)	67.1 (-3.0_200.8)	-1.93	**0.02**	0.52	139.7 (0.2_625.5)	65.6 (-4.5_313.5)	-2.13	**0.00**	**0.02**
2. Positive regulators of glycogen synthesis**													
**ATG7**	3p25.3	ILMN_ 1790978	164.0 ± 86.6	172.0 (4.2_392.6)	114.2 (27.2_335.6)	-1.51	**0.01**	0.29	130.5 (11.0_334.3)	168.6 (4.2_392.6)	1.29	0.29	1.00
**BAP1**	3p21.1	ILMN_ 1768363	87.2 ± 65.6	96.4 (-0.4_251.8)	56.2 (-1.0_184.2)	-1.72	**0.01**	0.13	65.9 (2.4_205.4)	89.9 (-1.0_251.8)	1.36	0.14	1.00
**EPM2A**	6q24.3	ILMN_ 1719468	34.1 ± 22.9	33.4 (-4.5_95.6)	24.7 (-7.6_63.7)	-1.35	0.09	1.00	37.1 (-4.5_95.6)	25.1 (-7.6_93.5)	-1.48	0.10	1.00
		ILMN_ 1788156	0.8 ± 4.3	0.9 (-4.2_22.2)	-0.7 (-5.9_5.4)	n.a.	**<0.05**	1.00	0.6 (-5.9_22.2)	0.1 (-4.2_7.4)	-6.00	0.35	1.00
INSR	19p13.2	ILMN_ 1670918	8.3 ± 8.8	7.0 (-1.6_20.7)	7.0 (-1.6_49.1)	1.00	0.85	1.00	5.7 (-1.4_18.1)	7.2 (-1.6_49.1)	1.26	0.71	1.00
IRS2	13q34	ILMN_ 2083469	531.3 ± 463.8	381.3 (58.9_1794.7)	309.6 (48.3_1461.4)	-1.23	0.82	1.00	366.2 (58.9_1696.2)	392.0 (48.3_1794.7)	1.07	0.71	1.00
IRS4	Xq22.3	ILMN_ 1712774	25.5 ± 37.1	17.8 (6.5_215.7)	14.4 (3.9_52.9)	-1.24	0.29	1.00	12.2 (6.5_215.7)	17.8 (3.9_199.8)	1.46	0.32	1.00
PPP1R3B	8p23.1	ILMN_ 2131103	-0.5 ± 3.5	-0.7 (-7.4_10.0)	-1.8 (-7.3_5.8)	n.a.	0.34	1.00	-1.3 (-7.4_10.0)	-1.3 (-7.3_6.3)	n.a.	0.87	1.00
**PPP1R3C**	10q23.32	ILMN_ 1736670	418.5 ± 507.6	352.4 (-0.7_2020.3)	85.9 (-1.6_2063.4)	-4.1	**0.01**	0.22	209.8 (21.7_1774.9)	155.6 (-1.6_2063.4)	-1.35	0.37	1.00
3. Negative regulators of glycogen synthesis**													
CALM1	14q32.11	ILMN_ 1778242	1194.4 ± 611.7	1013.2 (457.7_2604.7)	987.3 (422.5_2908.2)	-1.03	1.00	1.00	1077.5 (457.7_2908.2)	865.3 (422.5_2055.0)	-1.25	0.10	1.00
CALM2	2p21	ILMN_ 1687858	2341.5 ± 1318.3	2381.2 (677.6_4147.6)	2303.9 (536.1_6124.7)	-1.03	0.71	1.00	2043.8 (708.3_4479.9)	2468.1 (536.1_6124.7)	1.21	0.33	1.00
G6PC3	17q21.31	ILMN_ 2127477	467.7 ± 265.9	395.5 (37.9_1361.0)	425.2 (35.1_1120.1)	1.08	0.75	1.00	412.1 (133.3_1361.0)	412.9 (35.1_1120.1)	1.00	0.87	1.00

GFPT1	2p13.3	ILMN_ 2220187	241.9 ± 151.7	168.3 (63.5_770.3)	229.3 (137.1_931.7)	1.36	0.05	1.00	174.5 (110.8_393.6)	206.3 (63.5_931.7)	1.18	0.33	1.00
GSK3B	3q13.33	ILMN_ 1779376	525.6 ± 418.5	408.4 (88.1_1572.4)	528.1 (77.2_1942.5)	1.29	0.85	1.00	404.0 (88.1_1572.4)	563.3 (77.2_1942.5)	1.39	0.08	1.00
PCK1	20q13.31	ILMN_ 2197381	-2.7 ± 3.8	-3.9 (-9.9_5.9)	-2.4 (-9.0_5.8)	n.a.	0.45	1.00	-3.9 (-9.9_5.9)	-2.4 (-9.0_4.6)	n.a.	0.57	1.00
PHKA2	Xp22.13	ILMN_ 1814074	846.8 ± 643.3	623.7 (109.6_2289.3)	679.1 (167.3_2590.5)	1.09	0.80	1.00	567.1 (136.7_1946.6)	726.5 (109.6_2590.5)	1.28	0.11	1.00
PPP1R14C	6q25.1	ILMN_ 1664855	-0.4 ± 5.6	-2.4 (-7.8_14.7)	-0.5 (-8.3_24.7)	n.a.	0.40	1.00	-2.4 (-7.8_24.7)	-0.8 (-8.3_14.7)	n.a.	0.39	1.00
PYGM	11q13.1	ILMN_ 1720849	1.1 ± 4.6	0.4 (-5.0_13.0)	0.6 (-7.7_19.2)	1.50	0.97	1.00	0.2 (-7.7_7.3)	0.6 (-5.0_19.2)	3.00	0.45	1.00
4. Dual regulators of glycogen synthesis**													
PGM2	4p14	ILMN_ 1673543	98.2 ± 64.4	84.4 (24.6_265.5)	90.0 (1.1_318.3)	1.07	0.19	1.00	86.0 (1.1_254.9)	82.0 (24.3_318.3)	-1.05	0.94	1.00
PGM5	9q21.11	ILMN_ 2271149	2.5 ± 9.1	1.1 (-6.6_47.8)	-0.1 (-5.8_37.7)	n.a.	0.09	1.00	0.9 (-5.1_37.7)	0.3 (-6.6_47.8)	-3.00	0.09	1.00
		ILMN_ 1781388	9.3 ± 19.2	3.6 (-4.0_107.0)	3.0 (-4.1_78.9)	-1.20	0.78	1.00	3.8 (-4.1_30.9)	2.6 (-3.2_107.0)	-1.46	0.95	1.00
		ILMN_ 1709590	16.8 ± 21.7	10.0 (-3.6_115.2)	8.8 (0.2_44.9)	-1.14	0.54	1.00	13.1 (-0.2_94.0)	7.4 (-3.6_115.2)	-1.77	0.28	1.00

GEO: Gene Expression Omnibus; n.a.: not analyzed; TCGA: The Cancer Genome Atlas; UM: Uveal melanoma.

Multiple probes were available for GYG2, EPM2A, and PGM5. Genes with a differential expression are indicated in bold.

*Fold changes were calculated only for the probes with positive median values after background substraction. Negative fold change values indicate the downregulation of genes.

**References for gene function provided in the main text. † Mann-Whitney U test. †† with Bonferroni correction.

cancers

© 2020 by the authors. Submitted for possible open access publication under the terms and conditions of the Creative Commons Attribution (CC BY) license (http://creativecommons.org/licenses/by/4.0/).
